# Emergency response to a cluster of suspected food-borne botulism in Abuja, Nigeria: challenges with diagnosis and treatment in a resource-poor setting

**DOI:** 10.11604/pamj.2020.36.287.20872

**Published:** 2020-08-17

**Authors:** Oyeladun Okunromade, Mahmood Muazu Dalhat, Aminatu Makarfi Umar, Augustine Olajide Dada, Jamilu Nikau, Lamin Maneh, Okokon Ita Ita, Muhammad Shakir Balogun, Patrick Nguku, Olubunmi Ojo, Chikwe Ihekweazu

**Affiliations:** 1 Nigeria Centre for Disease Control, Abuja, Nigeria,; 2 Nigeria Field Epidemiology and Laboratory Training Programme, Abuja, Nigeria

**Keywords:** Case report, botulism, anti-toxin, botulinum, mouse assay

## Abstract

Food-borne botulism is a rare, acute and potentially fatal neurologic disorder that results from ingestion of food contaminated by botulinum toxin released from the anaerobic, spore-forming, gram-positive bacterium Clostridium botulinum. We reported an unusual cluster of botulism outbreak with high case fatality affecting a family following ingestion of home-made fish. A suspected outbreak of botulism affecting three patients in a family of six was reported to the Nigeria Centre for Disease Control. A rapid response team investigated by line-listing all the family members, interviewed extended family members, caregivers, clinicians, and nurses to collect socio-demographic and clinico epidemiological information using a semi-structured questionnaires. We collected blood from patients and food samples and locally made drink from the family home for laboratory testing. All family members ingested the same home-made food within the 48hrs before onset of symptoms in the index case. The clinical presentation of the three affected cases (AR=50.0%) was consistent with botulinum poisoning. Two of the affected cases died (CFR=66.7%) within 48hrs of admission, before antitoxin was made available. The third case had a milder presentation and survived, after administration of appropriate antitoxin. The remaining three children developed no symptoms. None of the samples cultured Clostridium botulinum. The blood samples were negative for mouse lethality test. Our report describes the challenges of diagnosis and management of rare emerging infectious disease outbreaks in resource-constrained settings.

## Introduction

About one thousand cases of botulism are reported annually worldwide [1]. Detection and reporting of botulism is rare in Nigeria and thus, there is no national reporting system for botulism as it is not among the list of notifiable disease through the Integrated Disease Surveillance and Response (IDSR) strategy used in Nigeria. Botulism is a rare, acute and potentially fatal neurologic disorder caused by the toxin of anaerobic, spore-forming, gram-positive bacterium- *Clostridium botulinum*. The toxins are among the most of lethal known substances. Seven different types of botulinum toxin have been identified, namely A, B, C, D, E, F, and G, four of which (types A, B, E and rarely F) are known cause of disease in humans, with A being the most commonly incriminated in outbreaks, followed by types B and E [2, 3]. Botulism mostly manifests in three forms; foodborne botulism, infant botulism and wound botulism. Other rare forms of the disease include inhalation botulism, or as an adverse effect of “botox” a pharmaceutical product used for cosmetic and other clinical conditions [4]. Of all forms of botulism, foodborne botulism is the most predominant worldwide [5], and it has the greatest public health concern because of its epidemic potential [6]. Food-borne botulism results from ingestion of food that is contaminated by the bacterial botulinum toxins released from *Clostridium botulinum*. Production of botulinum toxin occurs only under special conditions which include an anaerobic, low-salt, low-acid environment, and sugar content. Canned foods, ill preserved fish and native foods are the major sources of intoxication in the world, because of creating anaerobic conditions that allow *Clostridium botulinum* spores to germinate [7]. Foods in which the toxins have been isolated include preserved vegetables, fresh fish that is fermented, salted, smoked or canned tuna [8, 9]. Symptoms of botulism manifest after an incubation period of 12 to 36 hours (range: 4 hours to 8 days) [10].

Clinical features of Botulism include marked weakness, dry mouth, visual disturbances, dysphagia, and difficulty in talking and may progress to respiratory paralysis and involvement of muscle of the lower body. The diagnosis of food-borne botulism is based on high index of suspicion, clinical findings of cranial nerve palsies, descending paralysis, and history of ingestion of foods known to harbor the organism [11]. Laboratory confirmation, rarely available in resource-constrained settings require the demonstration of botulinum neurotoxin (BoNT) in patients' stomach or intestinal contents, vomit or faeces, blood during the hyper-acute stage, or the ingested food [12]. Identification of botulinum neurotoxin however requires specialized laboratory expertise, facilities and reagents, which are not readily available in Nigeria. The ingested toxin can be demonstrated in serum or faeces in 40 to 44% of cases within 3 days of toxin ingestion, and in 15 to 23% of cases in specimens obtained after 3 days [13]. Due to the life-threatening nature of botulism, rapid diagnosis is necessary for successful management. Therapy consists of administration of botulism antitoxin and supportive care with intensive care and long-term ventilation, when indicated [14]. A full recovery is possible, but the disease can have a long and exhausting course, and patients may die from complications of intensive care medicine. Case fatality ratio is between 5-10% [15, 16]. Factors reducing the risk of death include early diagnosis, prompt antitoxin administration and appropriate intensive care [3, 12].

## Methods

We report the response by Nigeria Centre for Disease Control (NCDC) to a suspected outbreak of botulism. A suspected outbreak of botulism affecting three patients in a family of six was reported to the NCDC on the 9^th^ of January 2018 by a private health facility in Abuja, Federal Capital Territory (FCT) and a rapid response team was deployed immediately to investigate and support the response. We line-listed all the family members and used semi-structured questionnaires to interview the admitted patients, family members and caregivers, clinicians and nurses in order to collect socio-demographic and clinical information. We conducted 72 hours dietary recall for all members of the family. Blood from patients and food samples from remnant fresh fish soup and locally made drink from the family home were collected for laboratory testing. The family of six (parents and four children) had partaken of a home cooked meal of pounded yam with fresh fish pepper soup 36 hours prior to onset of symptoms in the first patient (Table 1). There were no other meals consumed by any of the affected members. The other three children were followed up closely but did not develop symptoms. Samples of the blood of the affected individuals were taken and of all food items found in the house, including remnants in the rubbish bins were taken for laboratory analysis.

**Table 1:** seventy-two hours dietary recall for case 2

Date	Breakfast	Lunch	Dinner	Others
04/01/2018	Didn´t remember	Didn´t remember	Didn´t remember	------
05/01/2018	Bread, egg and tea (at home)	Rice (Office)	Pounded yam and fish (home)	------
06/01/2018	Meat pie and tea (in the office)	Rice (Office)	Buffet (office dinner party)	------

## Results

### Case Presentation

**Figure 1:**
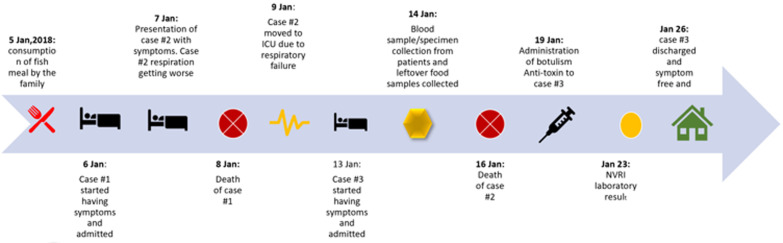
timeline for suspected botulism outbreak in Abuja, Nigeria, 2018

A 47-year-old female, P^4^, who resided at Ado in Karu, FCT, presented to a private clinic in Abuja on the 7^th^ of January, 2018 with vomiting, fever, sub-acute and progressive dizziness of about 8 hours duration which later progressed to generalized body weakness, dysphagia and odynophagia, dysarthria, left partial ptosis, sudden blurring vision, diplopia. Preliminary laboratory investigations conducted (full blood count, and electrolyte, Urea and Creatinine) were within normal range. Electorcardiogram investigation revealed sinus arrhythmia with intraventricular conduction block, and right atrial overload. Based on these findings initial diagnosis of ischaemic heart disease was made with oesophageal stricture and central retinal vein thrombosis. Within 24 hours of presentation her neurological symptoms worsened rapidly with bilateral ptosis and inability to open her eyes, and respiratory muscle paralyses followed by complete respiratory failure with progressive loss of consciousness. She died on the 8^th^ of January, 2018 (Table 2, Figure 1).

**Table 2:** disease course and outcome of cases

Case	Onset of symptoms	Symptoms onset to respiratory failure	Outcome
Case 1: female, age-47 (Mother)	7^th^ January,2018	<24hrs	Dead
Case 2: male, age-49 (Father)	7^th^ January, 2018	>24hrs	Dead
Case 3: Female, age-15 (daughter)	11^th^ January, 2018	Nil respiratory failure	Alive

The husband of case 1 presented to the same hospital on the same day with nausea, dizziness, vomiting, progressive dysarthria, odynophagia and partial ptosis of about 8 hours prior to presentation on the 7^th^ of January 2018. There was history of consumption of fish pepper soup prepared by case 1 the night prior to presentation, with ingredients sourced at the local market (see details 72 hours dietary recall in Table 1). Further information on the source of the fish, other ingredients and the method of preparation could not be ascertained. The meal was consumed by all members of the household. There was no history of consumption of canned foods or recreational drugs. Food recall could only be determined from Friday 5^th^ of January 2018. He spent the day at a symposium with his office colleagues during which there was a buffet at the office from which all his colleagues ate. The specific items he ate could not be ascertained, there was no ingestion of other snacks or in-between meals. He is a known hypertensive on treatment; not diabetic on any other long-term medication or suffering from other chronic illness. He was afebrile (axillary temperature (T) of 36.4°C), acyanosed, not jaundiced, with partial ptosis, pulse rate (PR) of 90 beats per minute (bpm), blood pressure (BP) of 150/98mmHg.

Except for serial reduction in platelet count (from 271x10^9^/L on the 8^th^ January to 83 x10^9^/L on the 9^th^ January 2018), full blood count, serum electorlyte, urea & creatinine, C reactive proteins, erythrocyte sedimentation rate, liver function tests, Urine toxicology for opiates, morphine, amphetamine, cocaine and other illicit drugs were normal. He had serial plasmapheresis and enema, was placed on polyvalent immunoglobulin (botulinum-specific immunoglobulin was unavailable), pyridostigmine, glycopyrolate, amlodipine, crystalline penicillin, and hydrocortisone. He deteriorated with complete ptosis, dysarthria and respiratory failure (SPO_2_=90%) within 8 hours of presentation. He was managed at the intensive care unit, where he was intubated, had percutaneous endoscopic gastrostomy (PEG), and placed on ventilatory support. He remained stable until day 5 when he developed tachycardia (PR of 120bpm) and uncontrolled hypertension (systolic BP of 180 to 220mmHg systolic and diastolic BP of 120 to 150 mmHg). He developed sudden cardiac arrest and died on the 7^th^ day on admission.

The daughter of the other two cases presented two days after the presentation of the first two cases with colicky abdominal pain, nausea, diplopia, dizziness and partial ptosis with intermittent headache. She was afebrile (T of 37.1°C), PR of 88 bpm, respiratory rate (RR) of 22 cycles per minute (cpm), BP of 113/61mmHg, and normal heart sounds. Similar investigations as done for case two turned out normal. She received polyvalent immunoglobulin (botulinum-specific immune The details of the food recall revealed that the family globulin was unavailable), repeated doses of activated charcoal and enema, and was placed on supportive therapy. Trivalent anti-toxin containing Type A, B and E ordered from Bangkok arrived on day seven after the onset. She had a stat dose of 250 ml slowly administered, followed 6 hours later with a continuous drip infusion of 250 ml. She was subsequently discharged after one week, and followed up for another one week. Apart from complains of mild dizziness, she reported no further symptoms post discharge.

The details of the food recall revealed that the family of six (parents and four children) had eaten a home-cooked meal of pounded yam with fresh fish pepper soup, a local delicacy, 36 hours prior to onset of symptoms in the first patient. The third case claimed eating only a small portion. The other three children were followed up closely but did not develop any symptoms.

**Laboratory diagnosis:** we collected blood from all the three cases for laboratory testing. Food samples from remnant fresh fish soup and locally made drink from the family home were also collected and sent to the laboratory for culture. The samples collected in this case series were transported in a geostyle box on ice packs, to the National Veterinary Research Institute (NVRI) Jos in Plateau State, Nigeria for preliminary testing. Duplicate samples were sent to the Centres for Disease Control and Prevention (CDC) Botulism Laboratory in Atlanta, USA. Samples were plated on enhanced medium and incubated anaerobically at 37°C for 24 hours for possible isolation of *clostridium* species. Colonial morphology and the gram stain reaction were not consistent with clostridia. The food and blood samples were used for toxins by intra-peritoneal injection of mice (the mouse lethality assay) diluted in phosphate buffer which were also negative after 72 hours.

## Discussion

We described an outbreak of suspected food-borne botulism in three members of a family of six with symptoms commencing 18 hours after consuming a home-prepared fish. The food-borne botulism was suspected based only on the typical signs and symptoms, such as cranial nerve paralysis including diplopia, blurred vision, ptosis, dysarthria, and dysphagia, followed by descending, symmetrical muscle paralysis, affecting the respiratory muscles at an early stage and on the information of ingestion of home-prepared fresh fish pepper soup before admission [1]. There is a clustering of cases in a family with a common food exposure. Despite the inability to get confirmed microbiological diagnosis of botulism in the described cluster, the typical clinical features, plausible history of fish ingestion, the commonest food substance implicated with botulism and the rapid response of the third case to antitoxin, make a strong case for a probable diagnosis of botulism in this outbreak. Though the disease can be fatal, the CFR of 66.7% in this outbreak was too high when compared more developed settings where documented CFR of foodborne botulism was 10-15% [17]. This might be the result of delayed clinical suspicion, inability to make prompt laboratory confirmation, and more importantly, delay in access to antitoxin. The fact that botulism is a rarely reported/diagnosed disease in our setting is likely to have contributed to the low index of suspicion. Few cases of the disease are reported in Africa; [18] and no known case has been previously reported in Nigeria. There is also limited capacity for the laboratory diagnosis and treatment of botulism in Nigeria due to competing priorities like viral hemorrhagic fevers, malaria, tuberculosis, Human immunodeficiency virus (HIV) infection, among others. Fish is one of the commonest foods linked to botulism [14]. We postulated that the fresh fish that was bought from a local market and temporarily stored for a day in the family refrigerator was contaminated with the botulism toxin. There have been reports of foodstuff contaminated with *Clostridium botulinum* from Nigerian markets [2].

The three suspected cases presented with common symptoms and signs of botulism, with case 1 manifesting more severe symptoms. She was initially managed as a case of ischemic heart disease. The correct suspected diagnosis was only made after her death when the husband's symptoms deteriorated. Late identification of the disease, non-administration of antitoxin and delay in commencement of intensive care might be responsible for the high case fatality seen in this outbreak. It has also been documented that autonomic dysfunction is one of the main complications of botulism especially type B [18] and the comorbidity in Case 2 (hypertensive) might be an additional factor for the poor outcome. As a result of its rare occurrence and its non-inclusion in Nigeria's surveillance system, most physicians have a low index of suspicion for prompt identification of botulism cases. These may be responsible for low index of clinical suspicion for botulism, lack of reporting, misdiagnosis of cases, unavailability of anti-toxin, very limited laboratory testing capacity for botulism and the accompanying unacceptable mortality. Non-availability of facilities, expertise and laboratory reagents to diagnose botulism in Nigeria is also a key challenge in effective response to the outbreak. Treatment was also a big challenge, as antitoxin was not available in Nigeria and could not be made available in time to save the second case, despite strong collaboration between NCDC and International partners who offered to help. The bioterrorism potential of the toxin contributed to the challenge of getting the required clearance to transfer it to the country to enable us save the lives of the two cases.

The response to botulism was limited by failure to isolate the organism or its toxins from either the cases or the food sample despite sending samples to different laboratories (both local and International) for testing. This was due to limited skill capacity on the type of samples to collect, the condition of sample collection, proper packaging and transportation. We were unable to get access to other family members to ascertain the amount of the fish they ingested and thus establish some dose-response relationship. Absence of symptoms in persons that consumed implicated food have also been reported [10]. We provided guidance to the clinicians managing the cases on the treatment protocols and monitored the patients progressively on a daily basis. We also prepared advisories for health workers and to the public on botulism. We recommend that institutions responsible for global health security, such as the World Health Organization should support the strengthening of specific public health reference laboratories in developing countries to develop the capacity for effective diagnosis of botulism. Arrangements can also be made to pre-position some antitoxin in developing countries to forestall future delay in diagnosis and treatment, and consequently high morbidity and mortality. Clinicians need to be sensitized on botulism detection and reporting through the routine surveillance system. The public also needs to be regularly informed on ways of avoiding commonly implicated sources of the organism.

## Conclusion

Although rarely reported in Africa and none reported in Nigeria prior to this incidence, this is a plausible outbreak of food-borne botulism from home prepared fish. The high case fatality recorded underscores the need for building human capacity and equipping laboratory to ensure timely diagnosis and treatment. Public health authorities should make antitoxin available to forestall future mortality from botulism and establish a clear line on anti-toxin supply.

### What is known about this topic

- There are usually high case fatality rate with cases of botulism;
- Where patients survive, they end up with a long term severe morbidity;
- Botulism anti-toxins are not easily and readily available in Africa.

### What this study adds

- The study draws attention of the global health world of the need to address the poor capacity of the developing countries to timely detect and effectively respond to botulism outbreaks based on the principles of the global health security agenda;
- Challenges of prompt laboratory diagnosis in Africa;
- Unavailability of botulism anti-toxin for prompt management to reduce the illness timelines.
